# Correlation between change in central subfield thickness and change in visual acuity in macular edema due to retinal vein occlusion: post hoc analysis of COPERNICUS, GALILEO, and VIBRANT

**DOI:** 10.1007/s00417-022-05697-4

**Published:** 2022-06-24

**Authors:** Sophie Z. Gu, Onnisa Nanegrungsunk, Susan B. Bressler, Weiming Du, Fouad Amer, Hadi Moini, Neil M. Bressler

**Affiliations:** 1 Johns Hopkins University School of Medicine, Baltimore, MD USA; 2 Retina Division, Wilmer Eye Institute, Johns Hopkins University School of Medicine, Baltimore, MD USA; 3 Retina Division, Department of Ophthalmology, Faculty of Medicine, Chiang Mai University, Chiang Mai, Thailand; 4 Regeneron Pharmaceuticals, Inc, Tarrytown, NY USA; 5 Department of Ophthalmology, Johns Hopkins University School of Medicine and Hospital, Maumenee 752, 600 N. Wolfe St, Baltimore, MD 21287-9277 USA

**Keywords:** Central subfield thickness, Macular edema, Retinal vein occlusion, Visual acuity

## Abstract

**Purpose:**

Assess correlation between change in central subfield thickness (CST) and change in best-corrected visual acuity (BCVA) in eyes with macular edema due to retinal vein occlusion (RVO) that received intravitreal aflibercept injections (IAI).

**Methods:**

Post hoc analysis of COPERNICUS and GALILEO trials for CRVO and VIBRANT trial for BRVO with relationships determined using Pearson correlation coefficient.

**Results:**

In COPERNICUS, correlations (r) between change in CST and change in BCVA from baseline at weeks 12, 24, 52, and 100 were −0.36 (95% CI: −0.52, −0.18; *P* < 0.001), −0.38 (95% CI: −0.53, −0.20; *P* < 0.001), −0.44 (95% CI: −0.58, −0.27; *P* < 0.001), and −0.41 (95% CI: −0.56, −0.23; *P* < 0.001), respectively. CST changes accounted for only 21% of the variance in BCVA changes; every 100-µm decrease in CST was associated with a 2.1-letter increase in BCVA (*P* = 0.003). Similar findings were noted for GALILEO (r, −0.45 to −0.23) and VIBRANT (r, −0.36 to −0.32) trials.

**Conclusion:**

In eyes treated with IAI for macular edema due to RVO, correlation between change in CST and change in BCVA was weak to moderate. While change in CST may be helpful in determining the need for anti-VEGF therapy, these findings do not support using changes in CST as a surrogate for changes in visual acuity outcomes. 
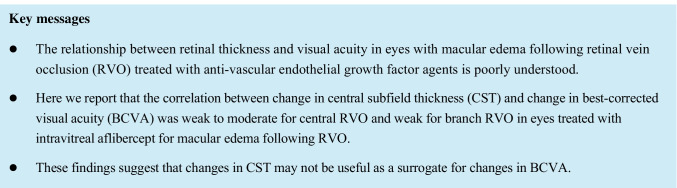

**Supplementary Information:**

The online version contains supplementary material available at 10.1007/s00417-022-05697-4.

## Introduction

Objective parameters, such as central subfield thickness (CST) noted on optical coherence tomography (OCT), have been suggested as biomarkers for predicting visual outcomes in retinal diseases. In eyes with retinal vein occlusion (RVO) with macular edema treated with anti-vascular endothelial growth factor (anti-VEGF) agents, there have been conflicting conclusions regarding whether CST correlates with visual acuity (VA), potentially related to limitations or interpretations of methods used. Previous studies have also shown mixed results regarding the prognostic value of baseline CST for visual outcomes [1–5]. Fewer studies have evaluated the correlation between change in CST and change in VA, and findings have been similarly mixed. In a prospective study of eyes with RVO treated with bevacizumab, Hoeh et al. showed a moderate correlation between change in central retinal thickness (CRT) and change in VA in branch RVO (BRVO), but none in central RVO (CRVO) [2]. Spaide et al. also observed no correlation between change in CRT and change in VA in a prospective case series of eyes with CRVO treated with ranibizumab [6]. In contrast, in a retrospective analysis of clinical trial data of eyes with RVO treated with ranibizumab, Ou et al. showed a moderate correlation between change in CRT and change in VA in CRVO, but none in BRVO [7]. Each of these studies had several limitations, including the following: (1) small numbers of participants which could limit the ability to identify correlations, (2) little, if any, standardization of refraction or visual acuity measurements, (3) little standardization or adherence to visit or treatment regimens, and (4) no data using fixed dosing regimens.

The correlation between change in CST and change in VA in eyes with macular edema due to RVO treated with anti-VEGF agents has not been examined extensively, to our knowledge, in large randomized clinical trials and may provide guidance for management. This investigation aimed to assess this correlation in a post hoc analysis of eyes with macular edema due to RVO treated with intravitreal aflibercept injections (IAI) from three randomized clinical trials. These included the COPERNICUS and GALILEO clinical trials, in which eyes with macular edema due to CRVO received IAI over 100 weeks (COPERNICUS) or 76 weeks (GALILEO), as well as the VIBRANT clinical trial, in which eyes with macular edema due to BRVO received IAI over 52 weeks.

## Methods

### Study designs of COPERNICUS, GALILEO, and VIBRANT

All three RVO studies were phase 3, randomized, double-masked, multicenter clinical trials. COPERNICUS (ClinicalTrials.gov identifier no. NCT00943072) was conducted across 61 sites in the USA, Canada, Colombia, India, and Israel. GALILEO (ClinicalTrials.gov identifier no. NCT01012973) was carried out at 63 sites across Europe and Asia/Pacific. VIBRANT (ClinicalTrials.gov identifier no. NCT01521559) was conducted at 58 sites in North America and Japan [8–10]. All respective institutional review boards/ethics committees approved the study protocols, which were carried out in compliance with ethical guidelines of the Declaration of Helsinki, the International Conference on Harmonization guidelines for Good Clinical Practice, and, for US patients, the Health Insurance Portability and Accountability Act of 1996. All participants provided written informed consent before the initiation of the study-specific procedures [8–10]. This post hoc analysis was designed starting October 1, 2020, and completed on January 31, 2021, with IRB agreement from the Johns Hopkins University School of Medicine that the human subject research was exempt as it only used de-identified data from clinical trial participants whose written informed consent document, captured during the original informed consent process, included permission for using de-identified information for post hoc analyses like this investigation.

The design and eligibility criteria for all three trials have been reported previously. [8–10] Only one eye from each patient was included in these studies. COPERNICUS and GALILEO were 100-week and 76-week studies, respectively, that randomized patients with macular edema secondary to CRVO in a 3:2 ratio to receive IAI 2 mg (IAI 2q4) or sham injections every 4 weeks through week 24, for a total of six doses. [8, 9]

In COPERNICUS, from weeks 24 to 52, all study participants were evaluated monthly and received IAI pro re nata (PRN) based on prespecified re-treatment criteria [8]. From weeks 52 to 100, study participants from both study arms were evaluated at least quarterly and received IAI PRN according to the same re-treatment criteria. Participants could be evaluated and dosed as frequently as every 4 weeks if deemed necessary by the investigators.

In GALILEO, from weeks 24 to 48, patients in the IAI 2q4 group were evaluated every 4 weeks and received IAI PRN if they met similar prespecified re-treatment criteria as in COPERNICUS. Participants in the sham group continued to receive sham at all scheduled visits through week 48. From weeks 52 to 76, patients in both intravitreal aflibercept (IAI 2q4/IAI PRN) and sham (sham/IAI PRN) groups were monitored every 8 weeks and received intravitreal aflibercept PRN according to the same prespecified re-treatment criteria. [9]

VIBRANT was a 52-week trial that enrolled patients with BRVO or hemi-retinal vein occlusion with foveal center-involved macular edema [10]. Study participants were randomized 1:1 to receive either laser at baseline or IAI 2q4 from baseline through week 24 followed by IAI every 8 weeks (2q8) through week 52. Starting from week 12, patients in both treatment groups could receive rescue treatment based on prespecified criteria [10]. Eyes in the IAI group eligible for rescue treatment received active laser at week 36. Eyes in the laser group, if eligible for rescue treatment before week 24, received one additional laser from week 12 to week 20 and, if eligible for rescue treatment after week 24, received IAI 2q8 after three initial monthly doses.

### Post hoc analysis

This post hoc analysis evaluated the association between CST and BCVA as well as the association between changes in CST and changes in BCVA from baseline in participants with macular edema secondary to RVO treated with sham, laser, or IAI. All participants who were in the full analysis set (defined as patients who were randomized and had baseline BCVA and at least one post-baseline BCVA assessment) and had BCVA and CST measurements at baseline were included in this analysis. CST was measured by time domain (TD)-OCT in study participants with CRVO in COPERNICUS and GALILEO studies and by spectral domain (SD)-OCT in participants with BRVO in VIBRANT.

### Statistical analyses

Data from COPERNICUS, GALILEO, and VIBRANT studies were analyzed separately. An initial analysis of data indicated that both absolute and changes in BCVA and CST showed a normal distribution across all treatment groups. Hence, Pearson correlation coefficient was used to evaluate the association between CST and BCVA at baseline and weeks 12, 24, 52, and 100 as well as between changes in CST and changes in BCVA at weeks 12, 24, 52, and 100 for COPERNICUS. Similar analyses were performed for GALILEO (at baseline and weeks 12, 24, 52, and 76) and VIBRANT (at baseline and weeks 12, 24, and 52). The 95% confidence intervals (CIs) of the Pearson correlation coefficients (r) were estimated using Fisher z transformation, and the relationship between the two variables was considered negligible for (r) from 0 to < 0.1, weak for (r) from 0.1 to < 0.40, moderate for (r) from 0.4 to < 0.7, strong for (r) from 0.7 to < 0.9, and very strong for (r) from 0.9 to 1 [11]. Generalized linear regression models were used to calculate the slope of the regression line for change in CST and the coefficient of determination (R^2^) for each visit. For all three trials, at the final study visit, previously identified baseline factors (age, BCVA, perfusion status, and time since diagnosis) that may be associated with BCVA changes and may be confounding variables were included in the models. Observed data were used at each time point. Data were censored after rescue medication was given. All *P* values reported were 2-sided, and no adjustments were made for multiplicity. Analyses were performed using SAS version 9.4 (SAS Institute, Inc., Cary, North Carolina).

## Results

### COPERNICUS

Of 187 eyes in the full analysis set (FAS), 114 eyes were randomized to receive IAI from the start of the trial. The percentage of eyes with both BCVA and CST measurements available for analysis at weeks 12, 24, 52, and 100 was 94%, 92%, 92%, and 88%. At baseline, the correlation (r) between CST and BCVA was −0.50 (95% CI: −0.63 to −0.34; *P* < 0.001). At weeks 12, 24, 52, and 100, the r values were −0.10 (95% CI: −0.28 to 0.10; *P* = 0.33), 0.19 (95% CI: −0.00 to 0.37; *P* = 0.05), −0.24 (95% CI: −0.41 to −0.05; *P* = 0.01), and −0.24 (95% CI: −0.42 to −0.05; *P* = 0.01), respectively (Table 1). Supplementary Figs. 1 and 2 show the linear regression between CST and VA at baseline and weeks 12, 24, 52, and 100 for the sham and IAI groups.

**Table 1:** Correlation between CST and BCVA by treatment group in COPERNICUS trial. In COPERNICUS, patients with macular edema secondary to CRVO received IAI 2q4 or sham injections every 4 weeks through week 24, for a total of six doses. From weeks 24 to 100, all study patients received IAI PRN (IAI + PRN and sham + IAI PRN) based on prespecified re-treatment criteria. *2q4*, 2 mg every 4 weeks; *BCVA*, best-corrected visual acuity; *CI*, confidence interval; *CST*, central subfield thickness; *IAI*, intravitreal aflibercept injection; *PRN*, pro re nata; *r*, correlation

Visit	Sham/IAI			IAI		
	*n*	r (95% CI)	*P-*value	*n*	r (95% CI)	*P-*value
Correlation between absolute CST and absolute BCVA						
Baseline	69	−0.43 (−0.60, −0.21)	0.0002	112	−0.50 (−0.63, −0.34)	< 0.001
Week 12	56	−0.34 (−0.55, −0.08)	0.01	107	−0.10 (−0.28, 0.10)	0.33
Week 24	55	−0.55 (−0.71, −0.33)	< 0.001	105	0.19 (−0.00, 0.37)	0.05
Week 52	56	0.07 (−0.20, 0.32)	0.63	105	−0.24 (−0.41, −0.05)	0.01
Week 100	49	−0.19 (−0.44, 0.10)	0.20	101	−0.24 (−0.42, −0.05)	0.01
Correlation between changes in CST and changes in BCVA						
Week 12	54	−0.20 (−0.44, 0.07)	0.15	106	−0.36 (−0.52, −0.18)	< 0.001
Week 24	52	−0.38 (−0.59, −0.11)	0.006	104	−0.38 (−0.53, −0.20)	< 0.001
Week 52	54	0.09 (−0.19, 0.35)	0.53	104	−0.44 (−0.58, −0.27)	< 0.001
Week 100	48	−0.03 (−0.31, 0.26)	0.86	100	−0.41 (−0.56, −0.23)	< 0.001

**Fig. 1:**
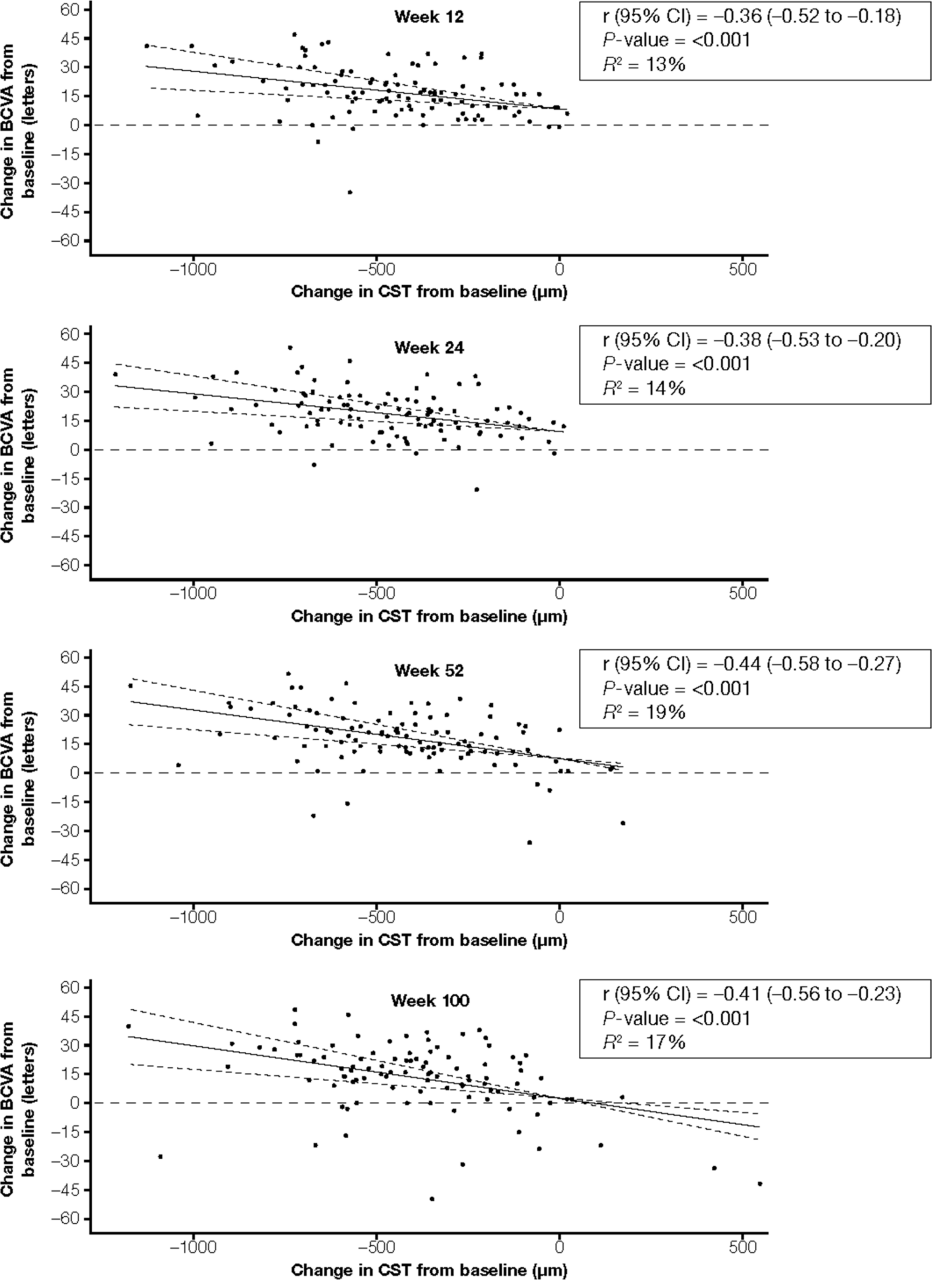
Correlations between changes in CST and changes in BCVA from baseline in the IAI group in the COPERNICUS trial. Solid lines indicate the correlation line, and dashed lines indicate the 95% CIs. In COPERNICUS, patients with macular edema secondary to CRVO received IAI 2q4 or sham injections every 4 weeks through week 24, for a total of six doses. From weeks 24 to 100, all study patients received IAI pro re nata (PRN) based on prespecified re-treatment criteria. 2q4, 2 mg every 4 weeks; 2q8, 2 mg every 8 weeks; BCVA, best-corrected visual acuity; CI, confidence interval; CST, central subfield thickness; IAI, intravitreal aflibercept injection; PRN, pro re nata; r, correlation; *R*.^*2*^, coefficient of determination

Correlations (r) between change in CST and change in BCVA from baseline at weeks 12, 24, 52, and 100 were −0.36 (95% CI: −0.52 to −0.18; *P* < 0.001), −0.38 (95% CI: −0.53 to −0.20; *P* < 0.001), −0.44 (95% CI: −0.58 to −0.27; *P* < 0.001), and −0.41 (95% CI: −0.56 to −0.23; *P* < 0.001), respectively (Table 1 and Fig. 1; see Supplementary Fig. 1, for the sham group). In a linear regression analysis of correlation between change in CST and change in BCVA at week 100 adjusted for baseline factors (age, perfusion status, time since diagnosis, and baseline BCVA), CST changes accounted for only 21% of the variance in BCVA changes. At 100 weeks, every 100-µm decrease in CST was associated with a 2.1-letter increase in BCVA; of note, however, the confidence interval was large (95% CI: 0.8 to 3.5; *P* = 0.003; Supplementary Table 1).

### GALILEO

Of 171 eyes in the FAS, 103 eyes were randomized to receive IAI from the start of the trial. The percentage of eyes with both BCVA and CST measurements available for analysis at weeks 12, 24, 52, and 76 was 93%, 94%, 86%, and 84%. At baseline, the correlation (r) between CST and BCVA was −0.23 (95% CI: −0.40, −0.04; *P* = 0.02). At weeks 12, 24, 52, and 76, the r values were −0.07 (95% CI: −0.26, 0.14; *P* = 0.52), −0.04 (95% CI: −0.24, 0.16; *P* = 0.70), −0.13 (95% CI: −0.33, 0.09; *P* = 0.24), and −0.52 (95% CI: −0.66, −0.35; *P* < 0.001), respectively (Supplementary Table 2).

Correlations (r) between change in CST and change in BCVA from baseline at weeks 12, 24, 52, and 76 for the IAI group were −0.30 (95% CI: −0.47 to −0.11; *P* = 0.003), −0.23 (95% CI: −0.41 to −0.03; *P* = 0.02), −0.40 (95% CI: −0.56 to −0.20; *P* = 0.001), and −0.45 (95% CI: −0.60 to −0.26; *P* < 0.001), respectively (Supplementary Table 2 and Figs. 4 through 7). In a linear regression analysis of correlation between change in CST and change in BCVA at week 76 adjusted for baseline factors (age, perfusion status, time since diagnosis, and baseline BCVA), CST changes accounted for only 33% of the variance in BCVA changes. At 76 weeks, every 100-µm decrease in CST was associated with a 2.4-letter increase in BCVA; of note, however, the confidence interval was large (95% CI: 1.3 to 3.5, *P* < 0.001; Supplementary Table 1).

### VIBRANT

Of 181 eyes in the FAS, 91 eyes were randomized to receive IAI from the start of the trial. The percentage of eyes with both BCVA and CST measurements available for analysis at weeks 12, 24, and 52 was 97%, 91%, and 80%. At baseline, the correlation (r) between CST and BCVA was −0.41 (95% CI: −0.56 to −0.22; *P* < 0.001). At weeks 12, 24, and 52, the r values were 0.13 (95% CI: −0.08 to 0.33; *P* = 0.22), 0.14 (95% CI: −0.08 to 0.35; *P* = 0.20), and 0.17 (95% CI: −0.06 to 0.39; *P* = 0.14), respectively (Table 2). Supplementary Figs. 8 and 9 show the linear regression between CST and VA at baseline and weeks 12, 24, and 52 for the laser/IAI and IAI groups.

**Table 2:** Correlation between CST and BCVA by treatment group in VIBRANT trial. In VIBRANT, patients with macular edema secondary to BRVO received either laser at baseline or IAI 2q4 from baseline through week 24. Both treatment groups received IAI 2q8 from week 24 through week 52. *2q4*, 2 mg every 4 weeks; *2q8*, 2 mg every 8 weeks; *BCVA*, best-corrected visual acuity; *CI*, confidence interval; *CST*, central subfield thickness; *IAI*, intravitreal aflibercept injection; *r*, correlation

Visit	Laser/IAI			IAI		
	*n*	r (95% CI)	*P-*value	*n*	r (95% CI)	*P-*value
Correlation between absolute CST and absolute BCVA						
Baseline	90	−0.46 (−0.61, −0.27)	< 0.001	91	−0.41 (−0.56, −0.22)	< 0.001
Week 12	83	−0.40 (−0.57, −0.20)	0.0002	88	0.13 (−0.08, 0.33)	0.22
Week 24	81	−0.46 (−0.62, −0.27)	< 0.001	83	0.14 (−0.08, 0.35)	0.20
Week 52	77	0.06 (−0.16, 0.28)	0.59	73	0.17 (−0.06, 0.39)	0.14
Correlation between changes in CST and changes in BCVA						
Week 12	83	−0.43 (−0.59, −0.24)	< 0.001	88	−0.34 (−0.51, −0.14)	0.001
Week 24	81	−0.42 (−0.59, −0.22)	< 0.001	83	−0.32 (−0.50, −0.11)	0.003
Week 52	77	−0.30 (−0.49, −0.08)	0.008	73	−0.36 (−0.54, −0.14)	0.002

**Fig. 2:**
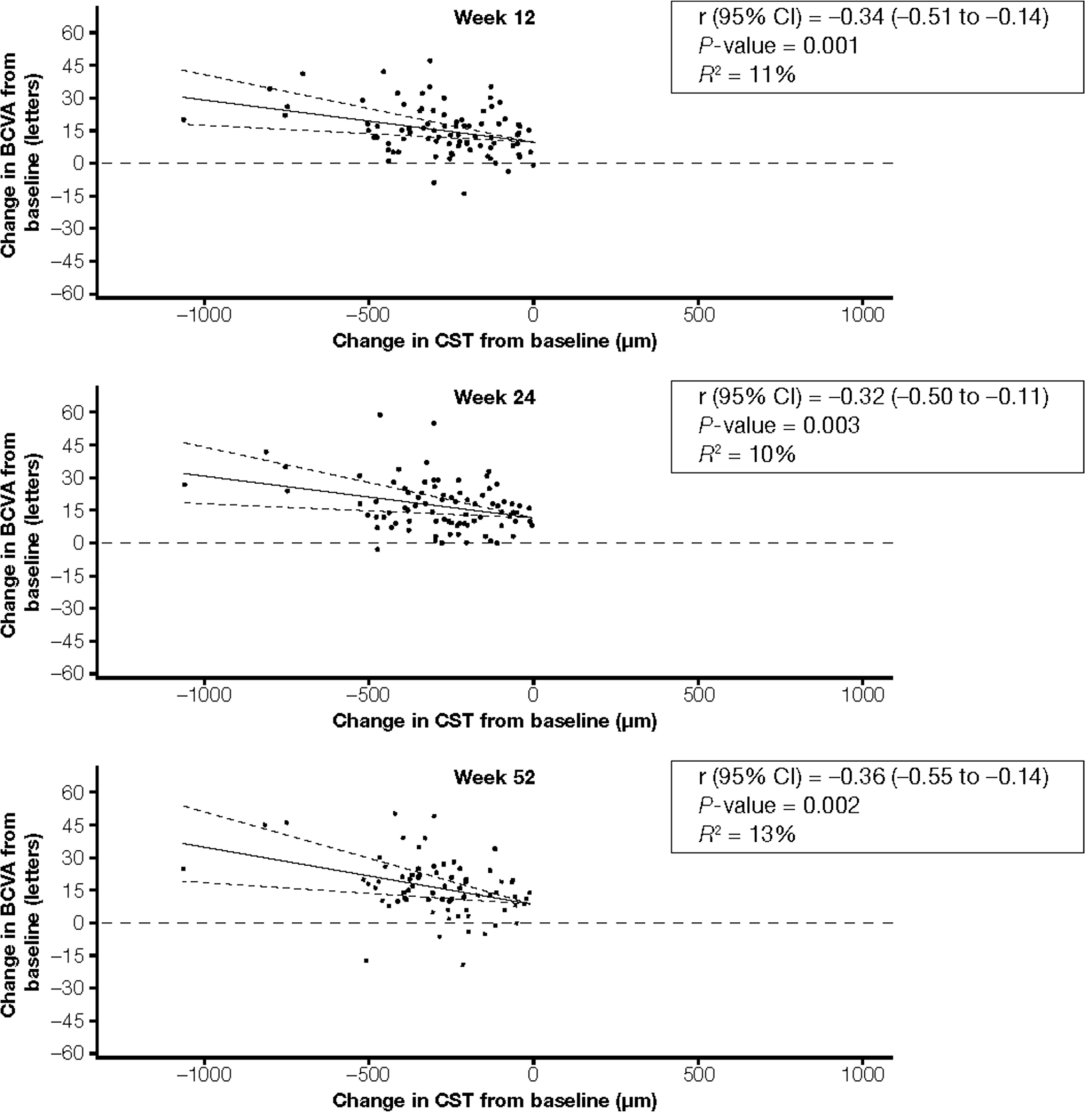
Correlations between changes in CST and changes in BCVA from baseline in the IAI group in the VIBRANT trial. Solid lines indicate the correlation line, and dashed lines indicate the 95% CIs. In VIBRANT, patients with macular edema secondary to BRVO received either laser at baseline or IAI 2q4 from baseline through week 24. Both treatment groups received IAI 2q8 from week 24 through week 52. 2q4, 2 mg every 4 weeks; 2q8, 2 mg every 8 weeks; BCVA, best-corrected visual acuity; CI, confidence interval; CST, central subfield thickness; IAI, intravitreal aflibercept injection; r, correlation; *R*.^*2*^, coefficient of determination

Correlations (r) between change in CST and change in BCVA from baseline at weeks 12, 24, and 52 were −0.34 (95% CI: −0.51 to −0.14; *P* = 0.001), −0.32 (95% CI: −0.50 to −0.11; *P* = 0.003), and −0.36 (95% CI: −0.54 to −0.14; *P* = 0.002), respectively (Table 2 and Fig. 2; see Supplementary Fig. 10). In a linear regression analysis of correlation between change in CST and change in BCVA at week 52 adjusted for baseline factors (age, perfusion status, time since diagnosis, and baseline BCVA), CST changes accounted for only 23% of the variance in BCVA changes. At 52 weeks, every 100-µm decrease in CST was associated with a 2.2-letter increase in BCVA; of note, however, the confidence interval was large (95% CI: −0.2 to 4.5; *P* = 0.07; Supplementary Table 1).

## Discussion

The findings of this study suggest that the association between CST and visual outcomes at baseline in eyes with macular edema due to RVO is weak to moderate for CRVO (r, −0.50 to −0.23) and moderate for BRVO (r, −0.41). These findings are comparable to those shown in the Standard Care Versus Corticosteroid for Retinal Vein Occlusion (SCORE) study, which reported a correlation coefficient of −0.27 for CRVO and −0.28 for BRVO [12]. In contrast, Ou et al. reported no correlation between CRT and VA at baseline in the Rubeosis Anti-VEGF (RAVE) and the Wide-field Angiography Guided Targeted Retinal Photocoagulation Combined with Anti-VEGF Intravitreal Injections for the Treatment of Ischemic Retinal Vein Occlusion (WAVE) trials [7].

Regarding the correlation between change in CST and change in VA from baseline at follow-up using a fixed-dosing regimen, the present study found a weak to moderate correlation (r, 0.45 to −0.23) for CRVO. Ou et al. reported a correlation coefficient of −0.43 at 12 months; similarly, the present study found correlation coefficients of −0.40 to −0.44 at week 52. By comparison, other studies have not been able to demonstrate a correlation between change in CST and change in VA in CRVO [3, 6]. The evidence for a correlation between change in CST and change in VA for BRVO is likewise mixed. The present study found a weak correlation (r, −0.36 to −0.32) between change in CST and change in VA for BRVO using a fixed-dosing regimen. Hoeh et al. reported a moderate correlation (r, 0.54) [3], while Ou et al. were not able to identify a correlation [7]. Although the results from these studies are varied, to our knowledge, no strong correlations have been found in recent publications in CRVO or BRVO; our investigation supports only weak or moderate correlations. This is consistent with results found in other diseases such as diabetic macular edema (DME) following standardized protocol refractions, visual acuity measurements, and monitored PRN regimens by the DRCR Retina Network [13, 14].

In linear regression analyses of correlation between change in CST and change in BCVA adjusted for baseline factors, only approximately a quarter (COPERNICUS and VIBRANT) to a third (GALILEO) of BCVA changes were attributable to CST changes, and every 100-µm decrease in CST was associated with only approximately a 2-letter gain in visual acuity. In conjunction with the weak to moderate correlations between change in CST and change in VA, these findings do not support the use of CST changes as a surrogate for VA changes in RVO, again similar to analyses done in DME by the DRCR Retina Network [13, 14].

This study has several strengths. This analysis included data from three relatively large randomized clinical trials conducted across multiple sites. Each trial included well-documented and consistent protocols for treatment, imaging, and VA testing. Eyes were included in the analysis only if they had verified BCVA and CST and adhered to the treatment regimen.

Limitations of this study include the post hoc design and thus inability to draw robust conclusions as the analyses was designed after the pre-planned primary, secondary, and exploratory outcomes were known. The analysis also includes CST only, without other qualitative or quantitative OCT or angiographic findings that may improve the predictive power of the linear regression model. To our knowledge, however, studies in other disease such as DME have not shown these other features to improve predictive power substantially beyond that provided by the baseline visual acuity and baseline OCT CST. Studies have reported, for instance, that anatomic features such as DRIL and/or EZ disruption on SD-OCT or the vascular density and foveal avascular zone area on OCT angiography may associate with visual acuity outcomes in eyes with RVO [15–18], but these studies were not very well controlled or did not involve a large number of patients with standardized protocols or treatment regiments. Additionally, while each trial followed a consistent protocol, differences between the protocols, such as specific treatment regimen, may affect the ability to compare results across trials.

In conclusion, in eyes treated with IAI for macular edema due to retinal vein occlusion, the correlation between change in CST and change in BCVA was weak to moderate for CRVO and weak for BRVO. While changes in CST may be important in determining the need for repeat anti-VEGF to manage macular edema due to retinal vein occlusion, these findings do not support using change in CST as a surrogate for change in visual acuity outcome.

## Supplementary Information

Below is the link to the electronic supplementary material.

Supplementary file1 (PDF 451 KB)

## Data Availability

Qualified researchers may request access to study documents (including the clinical study report, study protocol with any amendments, blank case report form, and statistical analysis plan) that support the methods and findings reported in this manuscript. Individual anonymised participant data will be considered for sharing once the indication has been approved by major health authorities and if there is legal authority to share the data and there is not a reasonable likelihood of participant re-identification. Submit requests to https://vivli.org/.
