# Supplementary material for: Correlation between change in central subfield thickness and change in visual acuity in macular edema due to retinal vein occlusion: post hoc analysis of COPERNICUS, GALILEO, and VIBRANT
Source: Graefes Arch Clin Exp Ophthalmol. 2022 Jun 24;260(12):3799–807. doi: 10.1007/s00417-022-05697-4 (PMC9666318; doi:10.1007/s00417-022-05697-4)
Supplement: Supplementary file 1 — Supplementary file1 (PDF 451 KB) [file 417_2022_5697_MOESM1_ESM.pdf]

Supplementary Material Contents

Supplementary Table 1. Effect of change in CST on the change in BCVA in patients treated with IAI 3

Supplementary Table 2. Correlation between CST and BCVA by treatment group in GALILEO trial 4

Supplementary Fig. 1 Correlations between absolute CST and absolute BCVA in the sham/IAI group in COPERNICUS trial........ 5

Supplementary Fig. 2 Correlations between absolute CST and absolute BCVA in the IAI group in COPERNICUS trial........ 8

Supplementary Fig. 3 Correlations between changes in CST and changes in BCVA from baseline in the sham/IAI group in COPERNICUS trial 11

Supplementary Fig. 4 Correlation between absolute CST and absolute BCVA in the sham/IAI group in GALILEO trial 13

Supplementary Fig. 5 Correlation between absolute CST and absolute BCVA in the IAI group in GALILEO trial........15

Supplementary Fig. 6 Correlations between changes in CST and changes in BCVA from baseline in the sham/IAI group in GALILEO trial 18

Supplementary Fig. 7 Correlations between changes in CST and changes in BCVA from baseline in the IAI group in GALILEO trial 20

Supplementary Fig. 8 Correlations between absolute CST and absolute BCVA in the Laser/IAI group in VIBRANT trial........22

Supplementary Fig. 9 Correlations between absolute CST and absolute BCVA in the IAI group in VIBRANT trial........24

Supplementary Fig. 10 Correlations between changes in CST and changes in BCVA in the Laser/IAI group in VIBRANT trial..26

Supplementary Table 1. Effect of change in CST on the change in BCVA in patients treated with IAI

Study	Visit	Change in CST (per 100 μm decrease)					
		n	Model Estimate	SE	95% CI	r^2	*P*-value
COPERNICUS	Week 100	99	2.1	0.7	(0.8, 3.5)	21%	0.003
GALILEO	Week 76	87	2.4	0.5	(1.3, 3.5)	33%	<0. 001
VIBRANT	Week 52	54	2.2	1.2	(-0.2, 4.5)	23%	0.07

BCVA, best-corrected visual acuity; CI, confidence interval; CST, central subfield thickness; IAI, intravitreal aflibercept injection; PRN, pro re nata; r, correlation; SE, standard error.

Supplementary Table 2. Correlation between CST and BCVA by treatment group in GALILEO trial

	Sham/IAI			IAI		
**Visit**	**n**	**r (95% CI)**	***P*-value**	**n**	**r (95% CI)**	***P*-value**
**Correlation between BCVA and CST**						
**Baseline**	68	–0.22 (–0.43, 0.03)	0.08	103	–0.23 (–0.40, –0.04)	0.02
**Week 12**	62	–0.40 (–0.59, –0.16)	0.001	96	–0.07 (–0.26, 0.14)	0.52
**Week 24**	55	–0.62 (–0.76, –0.42)	<0.001	97	–0.04 (–0.24, 0.16)	0.70
**Week 52**	50	–0.55 (–0.71, –0.31)	<0.001	89	–0.13 (–0.33, 0.09)	0.24
**Week 76**	50	–0.11 (–0.38, 0.18)	0.45	87	–0.52 (–0.66, –0.35)	<0.001
**Correlation between changes in BCVA and changes in CST**						
**Week 12**	62	–0.01 (–0.26, 0.24)	0.92	96	–0.30 (–0.47, –0.11)	0.003
**Week 24**	55	–0.34 (–0.55, –0.08)	0.01	97	–0.23 (–0.41, –0.03)	0.02
**Week 52**	50	–0.15 (–0.41, 0.14)	0.32	89	–0.40 (–0.56, –0.20)	<0.001
**Week 76**	50	0.13 (–0.15, 0.40)	0.35	87	–0.45 (–0.60, –0.26)	<0.001

In GALILEO, study participants with macular edema secondary to CRVO received IAI 2q4 or sham injections every four weeks through week 24, for a total of six doses. From weeks 24 to 48, study participants in the IAI 2q4 group received IAI PRN based on prespecified re-treatment criteria. Study participants in the sham group continued to receive sham through week 48. From week 52 to week 76, study participants in both groups received IAI PRN. 2q4, 2 mg every 4 weeks; 2q8, 2 mg every 8 weeks; BCVA, best-corrected visual acuity; CI, confidence interval; CST, central subfield thickness; IAI, intravitreal aflibercept injection; PRN, pro re nata; r, correlation.

Supplementary Fig. 1 Correlations between absolute CST and absolute BCVA in the sham/IAI group in COPERNICUS trial

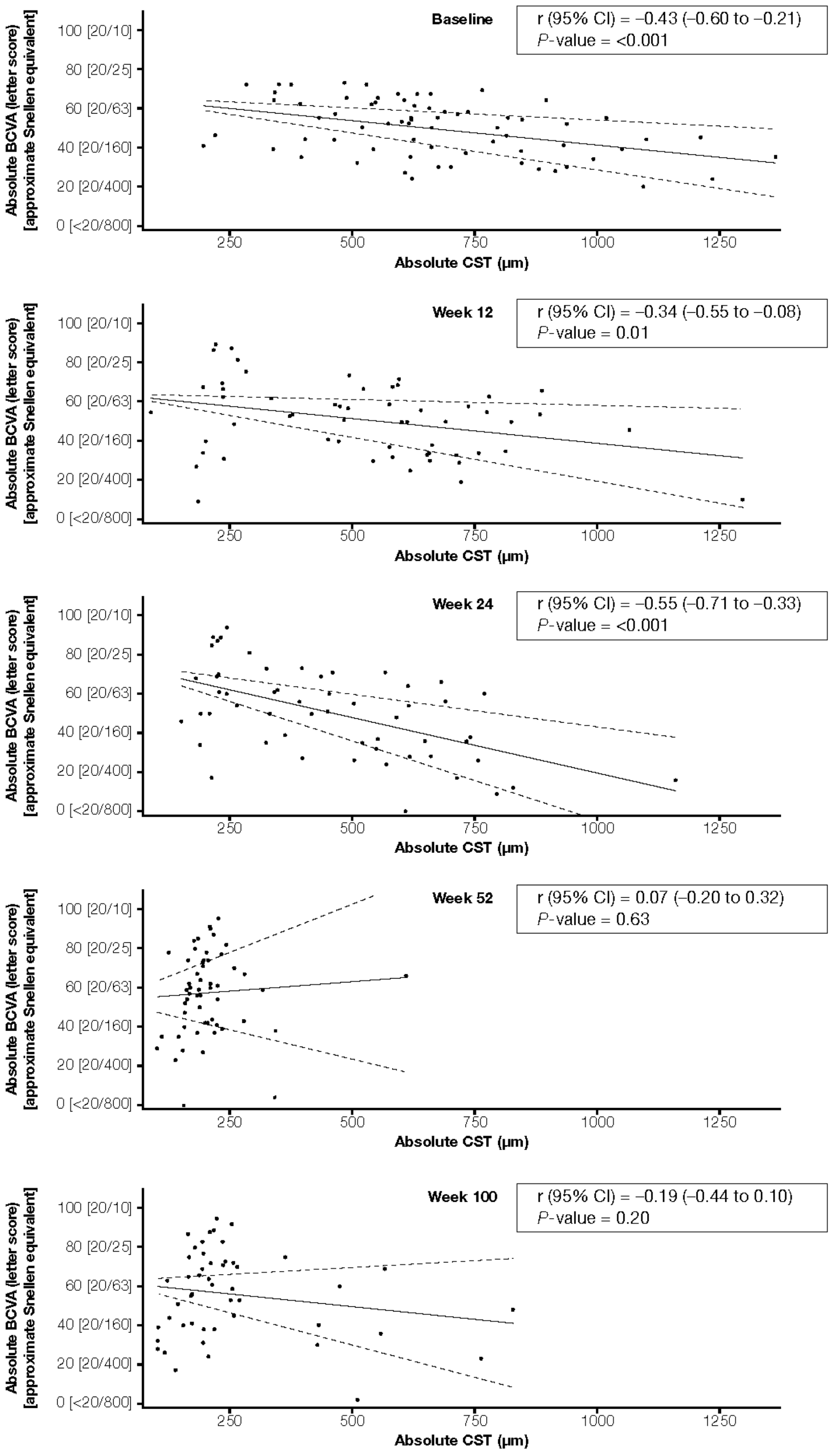
Baseline
r (95% CI) = −0.43 (−0.60 to −0.21)
P-value = <0.001
Week 12
r (95% CI) = −0.34 (−0.55 to −0.08)
P-value = 0.01
Week 24
r (95% CI) = −0.55 (−0.71 to −0.33)
P-value = <0.001
Week 52
r (95% CI) = 0.07 (−0.20 to 0.32)
P-value = 0.63
Week 100
r (95% CI) = −0.19 (−0.44 to 0.10)
P-value = 0.20
Absolute BCVA (letter score) [approximate Snellen equivalent]
100 [20/10]
80 [20/25]
60 [20/63]
40 [20/160]
20 [20/400]
0 [<20/800]
250
500
750
1000
1250
Absolute CST (μm)

Solid lines indicate the correlation line, and dashed lines indicate the 95% CIs. In COPERNICUS, study participants with macular edema secondary to CRVO received IAI 2q4 or sham injections every four weeks through week 24, for a total of six doses. From weeks 24 to 100, all study participants received IAI pro re nata (PRN) based on prespecified re-treatment criteria. 2q4, 2 mg every 4 weeks; BCVA, best-corrected visual acuity; CI, confidence interval; CST, central subfield thickness; IAI, intravitreal aflibercept injection; PRN, pro re nata; r, correlation.

Supplementary Fig. 2 Correlations between absolute CST and absolute BCVA in the IAI group in COPERNICUS trial

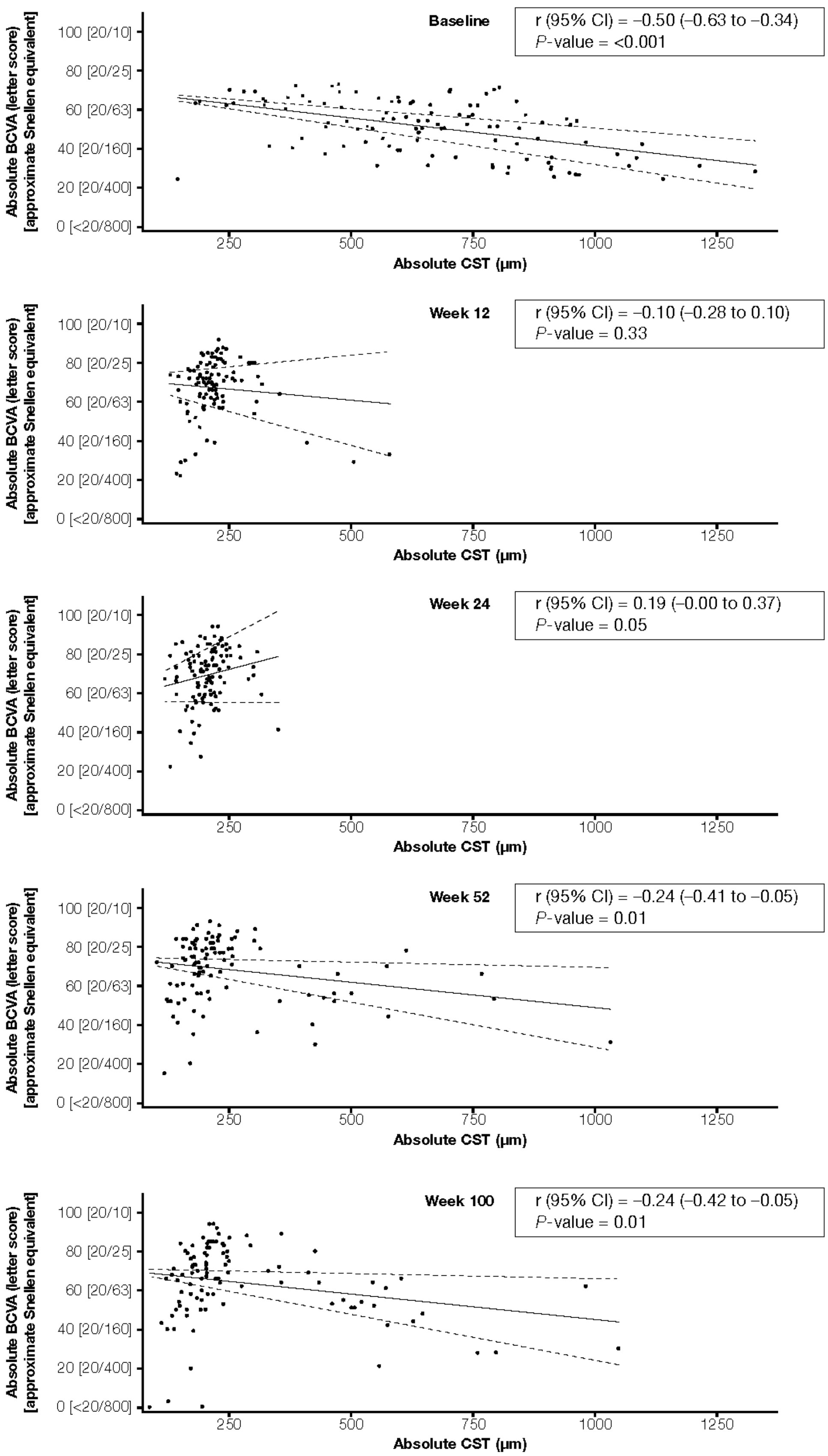
Baseline
r (95% CI) = –0.50 (–0.63 to –0.34)
P-value = <0.001
Week 12
r (95% CI) = –0.10 (–0.28 to 0.10)
P-value = 0.33
Week 24
r (95% CI) = 0.19 (–0.00 to 0.37)
P-value = 0.05
Week 52
r (95% CI) = –0.24 (–0.41 to –0.05)
P-value = 0.01
Week 100
r (95% CI) = –0.24 (–0.42 to –0.05)
P-value = 0.01
Absolute BCVA (letter score) [approximate Snellen equivalent]
100 [20/10]
80 [20/25]
60 [20/63]
40 [20/160]
20 [20/400]
0 [<20/800]
Absolute CST (µm)
250
500
750
1000
1250

Solid lines indicate the correlation line, and dashed lines indicate the 95% CIs. In COPERNICUS, study participants with macular edema secondary to CRVO received IAI 2q4 or sham injections every four weeks through week 24, for a total of six doses. From weeks 24 to 100, all study participants received IAI pro re nata (PRN) based on prespecified re-treatment criteria. 2q4, 2 mg every 4 weeks; BCVA, best-corrected visual acuity; CI, confidence interval; CST, central subfield thickness; IAI, intravitreal aflibercept injection; PRN, pro re nata; r, correlation.

Supplementary Fig. 3 Correlations between changes in CST and changes in BCVA from baseline in the sham/IAI group in COPERNICUS trial

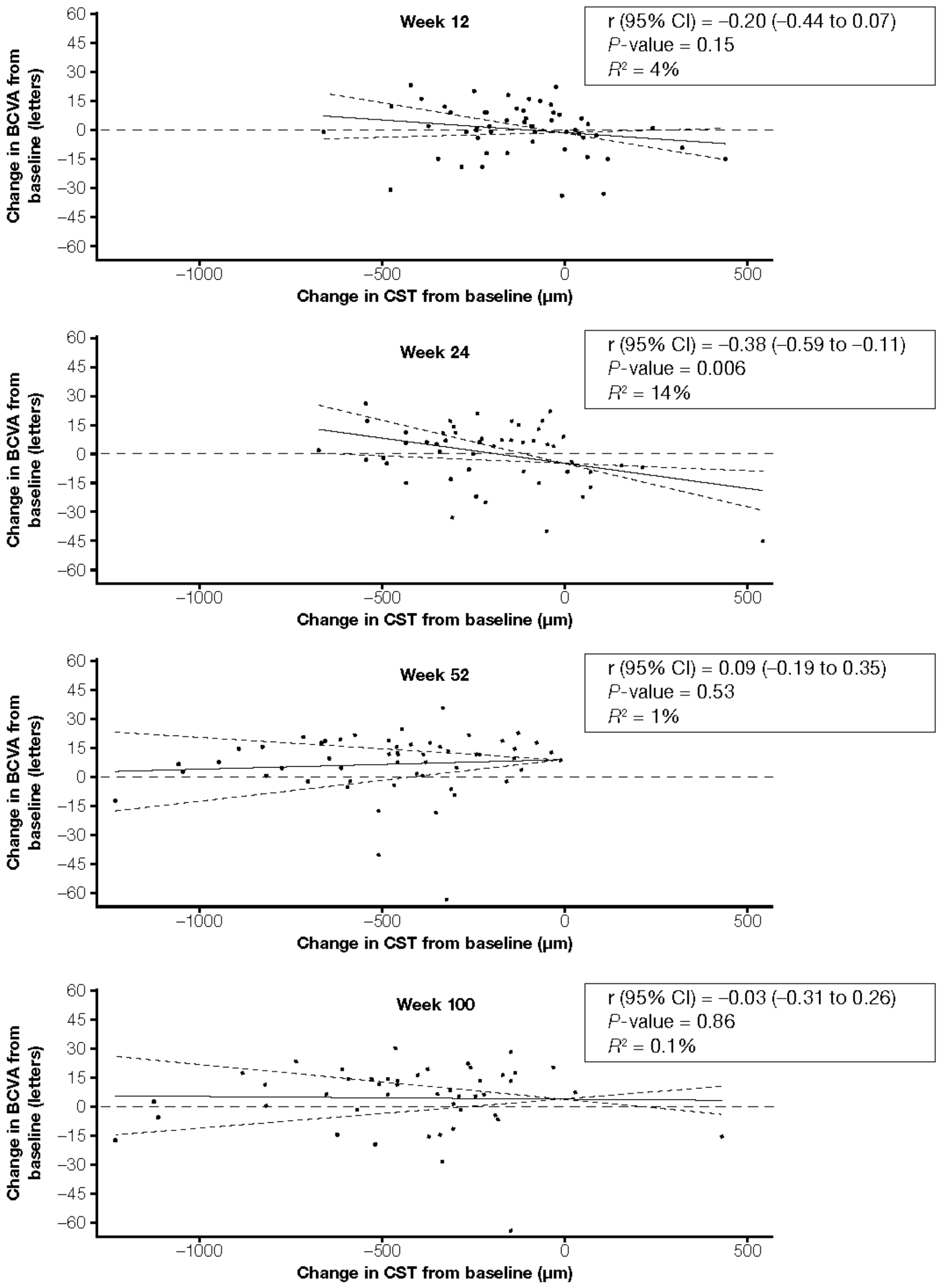

Solid lines indicate the correlation line, and dashed lines indicate the 95% CIs. In COPERNICUS, study participants with macular edema secondary to CRVO received IAI 2q4 or

sham injections every four weeks through week 24, for a total of six doses. From weeks 24 to 100, all study participants received IAI pro re nata (PRN) based on prespecified re-treatment criteria. 2q4, 2 mg every 4 weeks; BCVA, best-corrected visual acuity; CI, confidence interval; CST, central subfield thickness; IAI, intravitreal aflibercept injection; PRN, pro re nata; r, correlation; R^2, coefficient of determination.

Supplementary Fig. 4 Correlation between absolute CST and absolute BCVA in the sham/IAI group in GALILEO trial

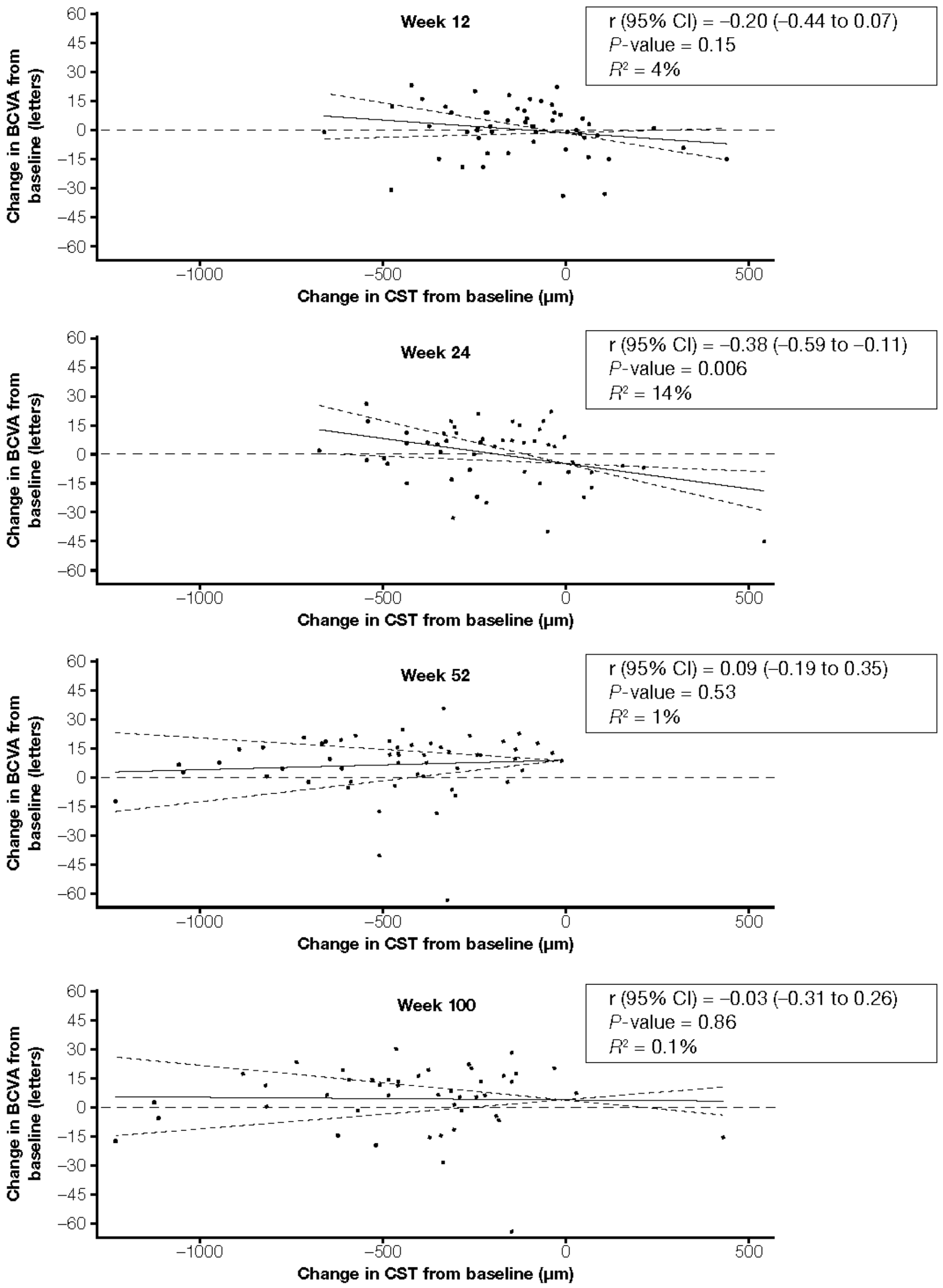

Solid lines indicate the correlation line, and dashed lines indicate the 95% CIs. In GALILEO, study participants with macular edema secondary to CRVO received IAI 2q4 or sham injections

every four weeks through week 24, for a total of six doses. From weeks 24 to 48, study participants in the IAI 2q4 group received IAI PRN based on prespecified re-treatment criteria. Study participants in the sham group continued to receive sham through week 48. From week 52 to week 76, study participants in both groups received IAI PRN. 2q4, 2 mg every 4 weeks; 2q8, 2 mg every 8 weeks; BCVA, best-corrected visual acuity; CI, confidence interval; CST, central subfield thickness; IAI, intravitreal aflibercept injection; PRN, pro re nata; r, correlation.

Supplementary Fig. 5 Correlation between absolute CST and absolute BCVA in the IAI group in GALILEO trial

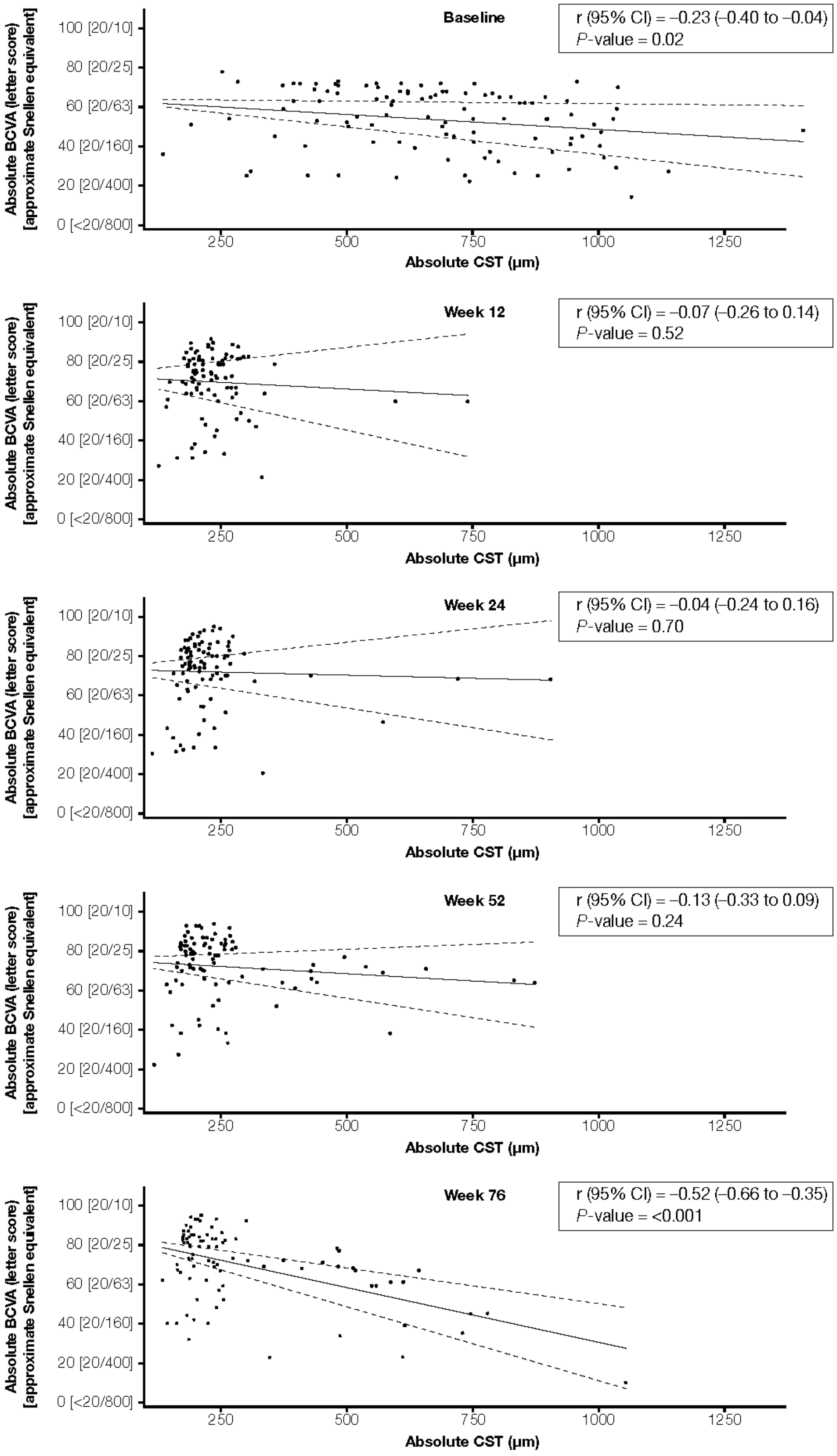
Baseline
r (95% CI) = −0.23 (−0.40 to −0.04)
P-value = 0.02
Week 12
r (95% CI) = −0.07 (−0.26 to 0.14)
P-value = 0.52
Week 24
r (95% CI) = −0.04 (−0.24 to 0.16)
P-value = 0.70
Week 52
r (95% CI) = −0.13 (−0.33 to 0.09)
P-value = 0.24
Week 76
r (95% CI) = −0.52 (−0.66 to −0.35)
P-value = <0.001
Absolute BCVA (letter score) [approximate Snellen equivalent]
100 [20/10]
80 [20/25]
60 [20/63]
40 [20/160]
20 [20/400]
0 [<20/800]
250
500
750
1000
1250
Absolute CST (µm)

Solid lines indicate the correlation line, and dashed lines indicate the 95% CIs. In GALILEO, study participants with macular edema secondary to CRVO received IAI 2q4 or sham injections every four weeks through week 24, for a total of six doses. From weeks 24 to 48, study participants in the IAI 2q4 group received IAI PRN based on prespecified re-treatment criteria. Study participants in the sham group continued to receive sham through week 48. From week 52 to week 76, study participants in both groups received IAI PRN. 2q4, 2 mg every 4 weeks; 2q8, 2 mg every 8 weeks; BCVA, best-corrected visual acuity; CI, confidence interval; CST, central subfield thickness; IAI, intravitreal aflibercept injection; PRN, pro re nata; r, correlation.

Supplementary Fig. 6 Correlations between changes in CST and changes in BCVA from baseline in the sham/IAI group in GALILEO trial

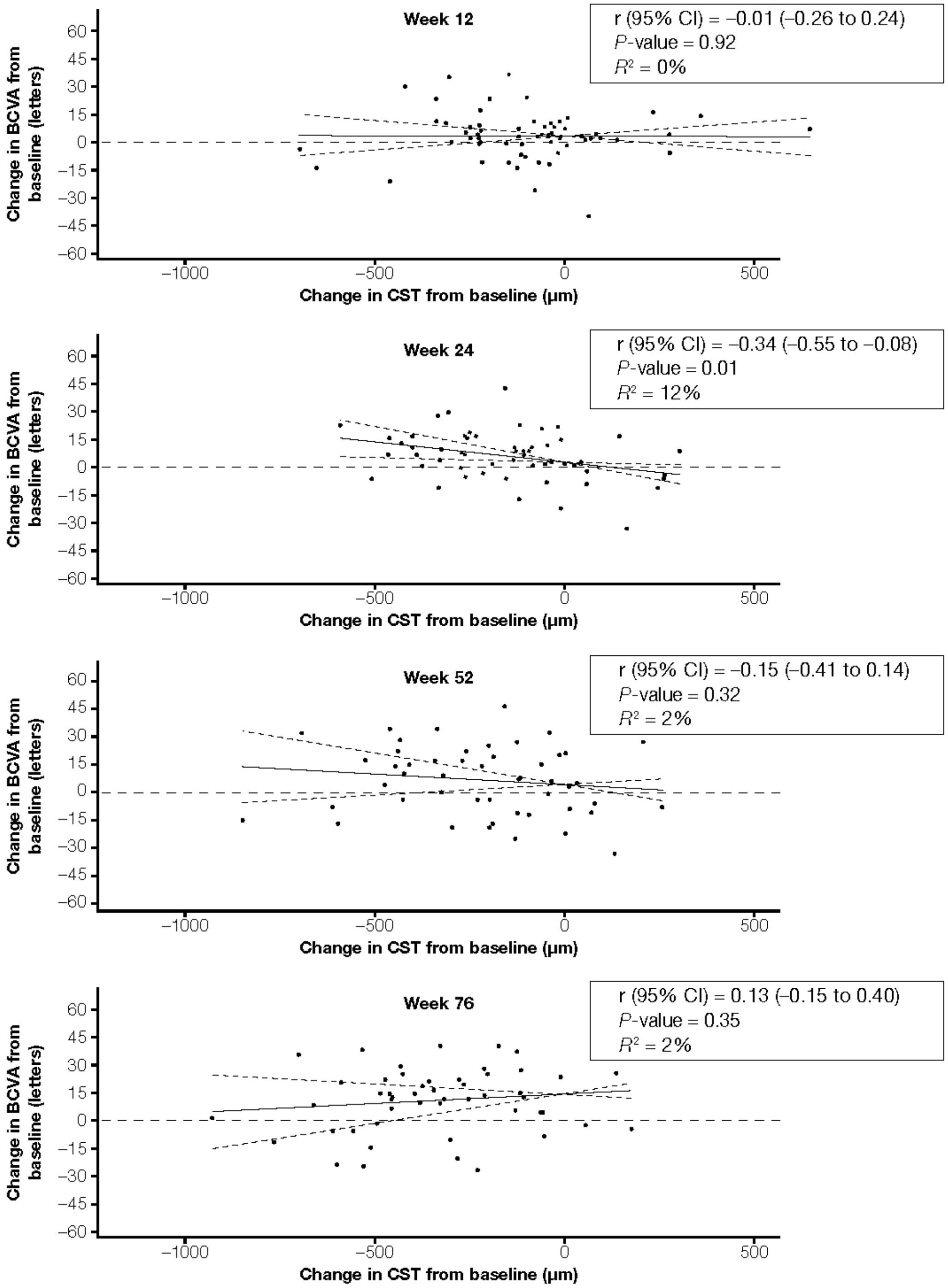

Solid lines indicate the correlation line, and dashed lines indicate the 95% CIs. In GALILEO, study participants with macular edema secondary to CRVO received IAI 2q4 or sham injections

every four weeks through week 24, for a total of six doses. From weeks 24 to 48, study participants in the IAI 2q4 group received IAI PRN based on prespecified re-treatment criteria. Study participants in the sham group continued to receive sham through week 48. From week 52 to week 76, study participants in both groups received IAI PRN. 2q4, 2 mg every 4 weeks; 2q8, 2 mg every 8 weeks; BCVA, best-corrected visual acuity; CI, confidence interval; CST, central subfield thickness; IAI, intravitreal aflibercept injection; PRN, pro re nata; r, correlation; R^2, coefficient of determination.

Supplementary Fig. 7 Correlations between changes in CST and changes in BCVA from baseline in the IAI group in GALILEO trial

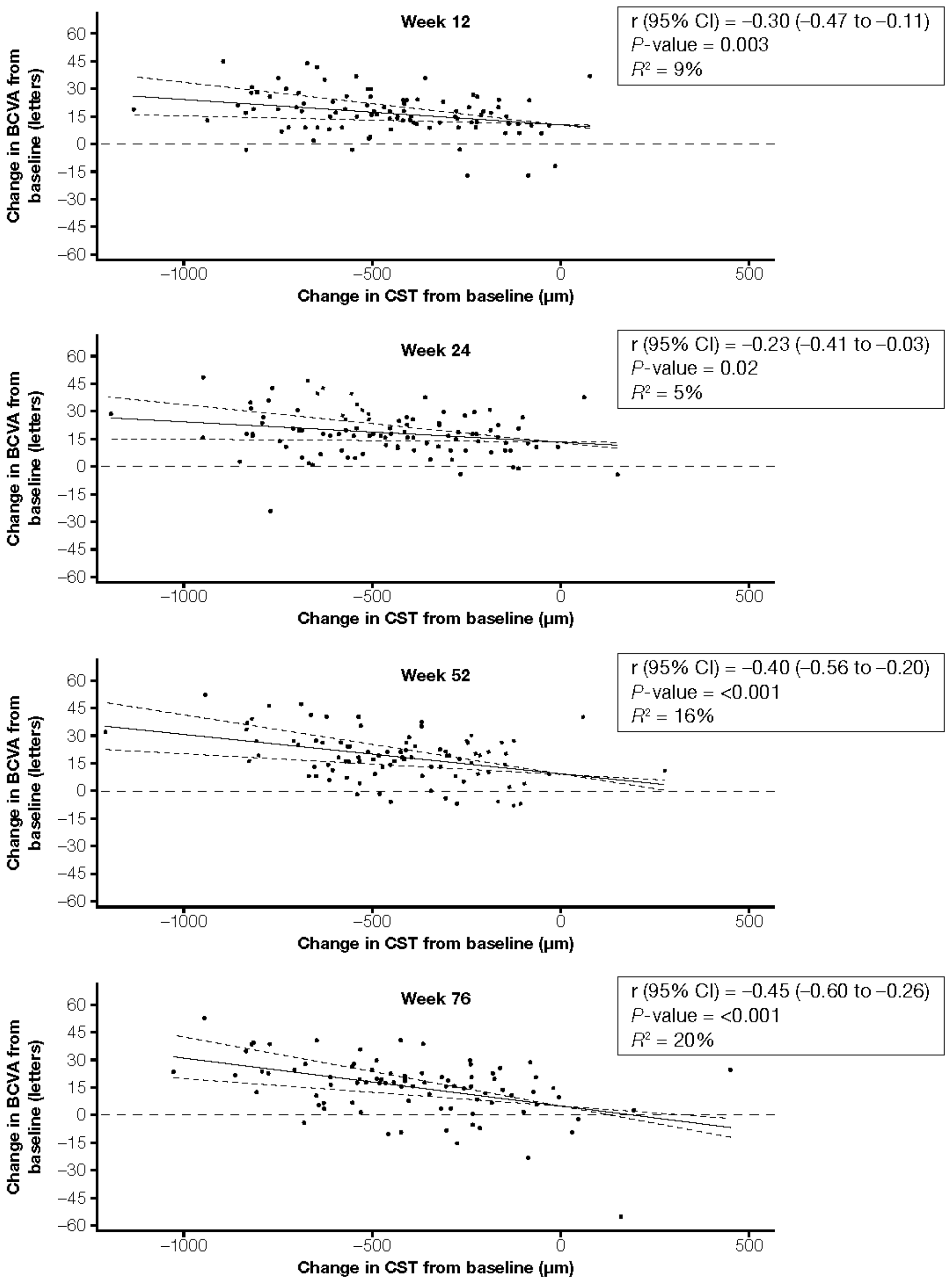

Solid lines indicate the correlation line, and dashed lines indicate the 95% CIs. In GALILEO, study participants with macular edema secondary to CRVO received IAI 2q4 or sham injections

every four weeks through week 24, for a total of six doses. From weeks 24 to 48, study participants in the IAI 2q4 group received IAI PRN based on prespecified re-treatment criteria. Study participants in the sham group continued to receive sham through week 48. From week 52 to week 76, study participants in both groups received IAI PRN. 2q4, 2 mg every 4 weeks; 2q8, 2 mg every 8 weeks; BCVA, best-corrected visual acuity; CI, confidence interval; CST, central subfield thickness; IAI, intravitreal aflibercept injection; PRN, pro re nata; r, correlation; R^2, coefficient of determination.

Supplementary Fig. 8 Correlations between absolute CST and absolute BCVA in the Laser/IAI group in VIBRANT trial

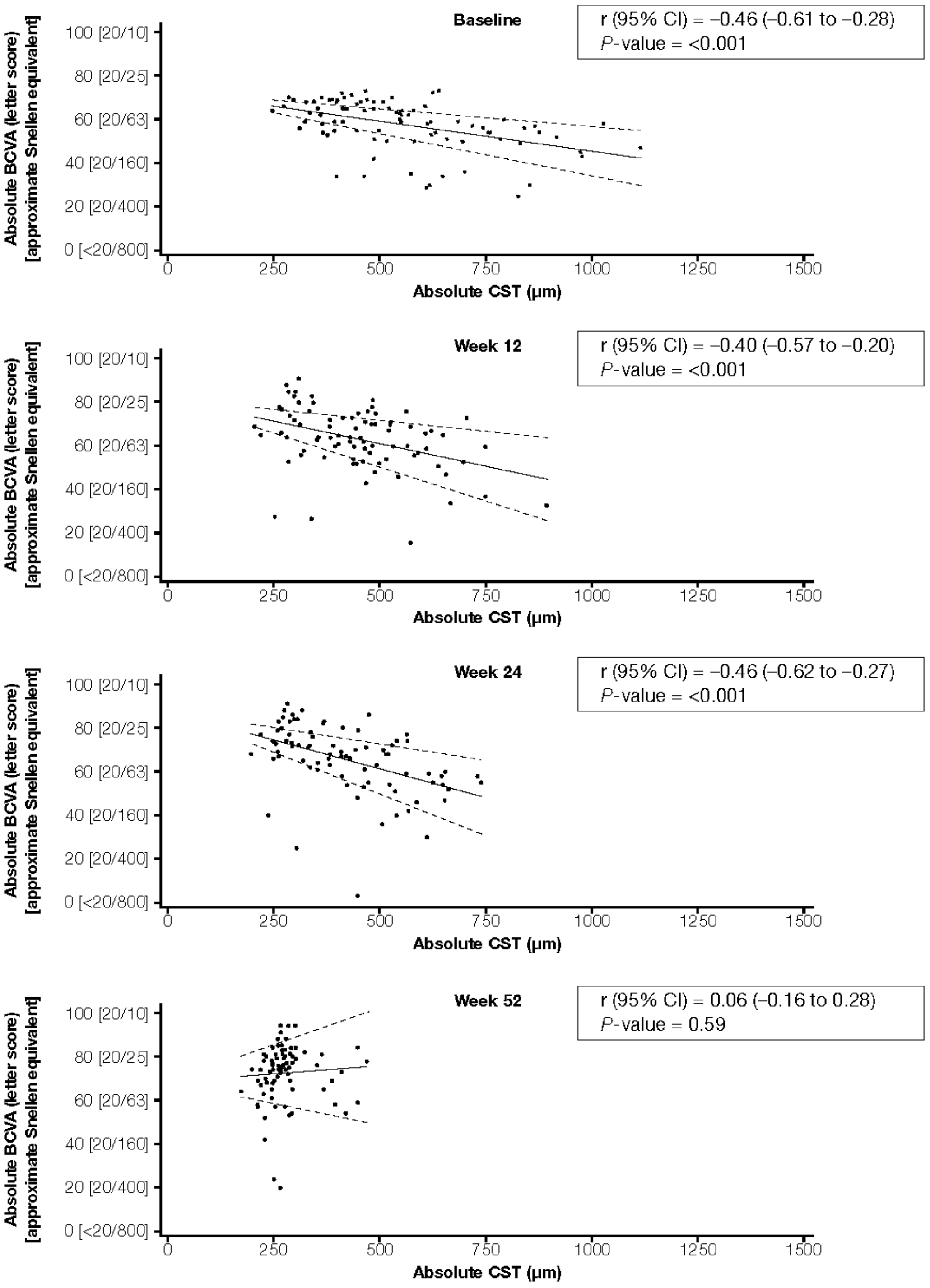

Solid lines indicate the correlation line, and dashed lines indicate the 95% CIs. In VIBRANT, study participants with macular edema secondary to BRVO received either laser at baseline or

IAI 2q4 from baseline through week 24. Both treatment groups received IAI 2q8 from week 24 through week 52. 2q4, 2 mg every 4 weeks; 2q8, 2 mg every 8 weeks; BCVA, best-corrected visual acuity; CI, confidence interval; CST, central subfield thickness; IAI, intravitreal aflibercept injection;

r, correlation.

Supplementary Fig. 9 Correlations between absolute CST and absolute BCVA in the IAI group in VIBRANT trial

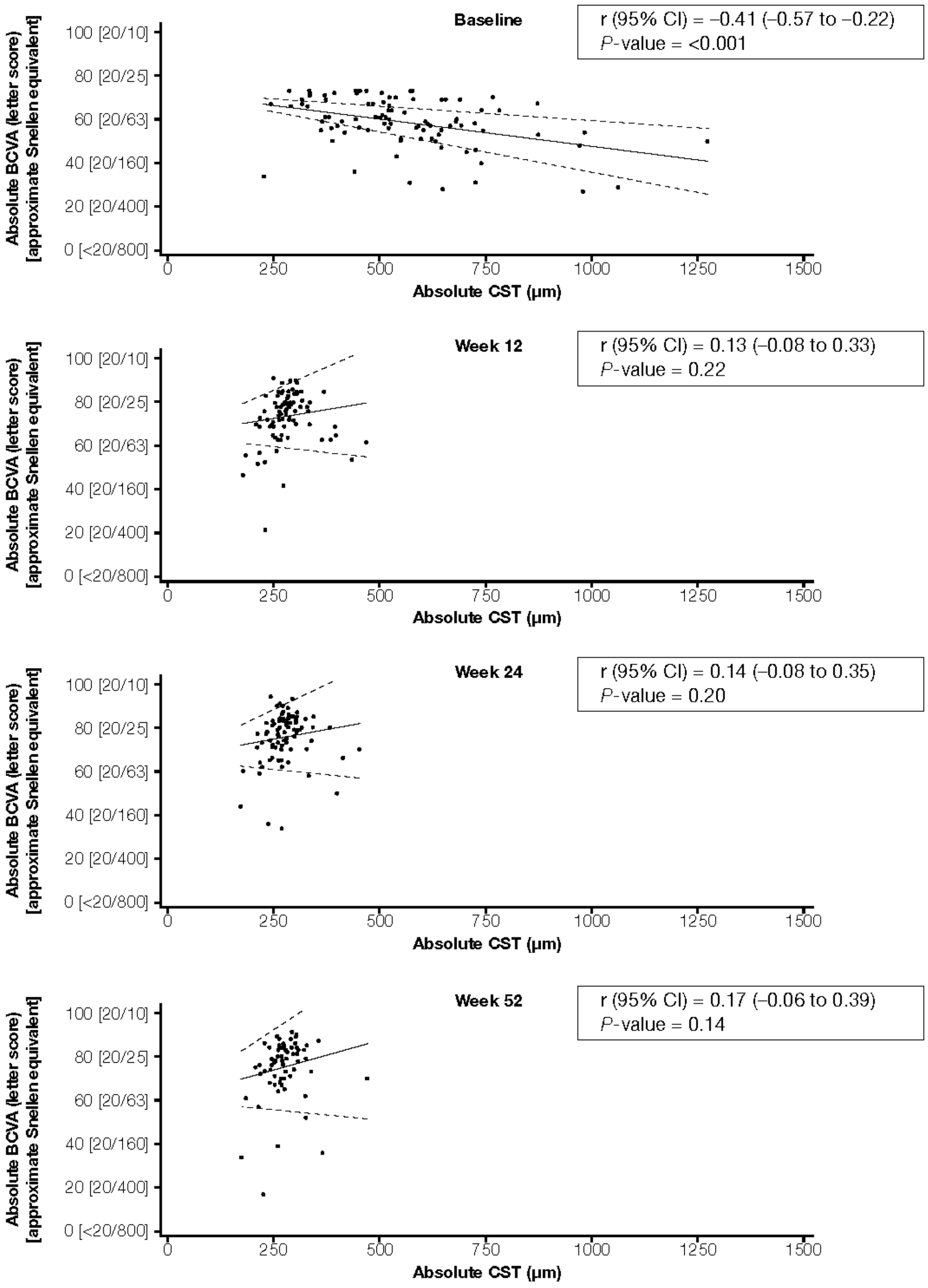

Solid lines indicate the correlation line, and dashed lines indicate the 95% CIs. In VIBRANT, study participants with macular edema secondary to BRVO received either laser at baseline or

IAI 2q4 from baseline through week 24. Both treatment groups received IAI 2q8 from week 24 through week 52. 2q4, 2 mg every 4 weeks; 2q8, 2 mg every 8 weeks; BCVA, best-corrected visual acuity; CI, confidence interval; CST, central subfield thickness; IAI, intravitreal aflibercept injection;

r, correlation.

Supplementary Fig. 10 Correlations between changes in CST and changes in BCVA in the Laser/IAI group in VIBRANT trial

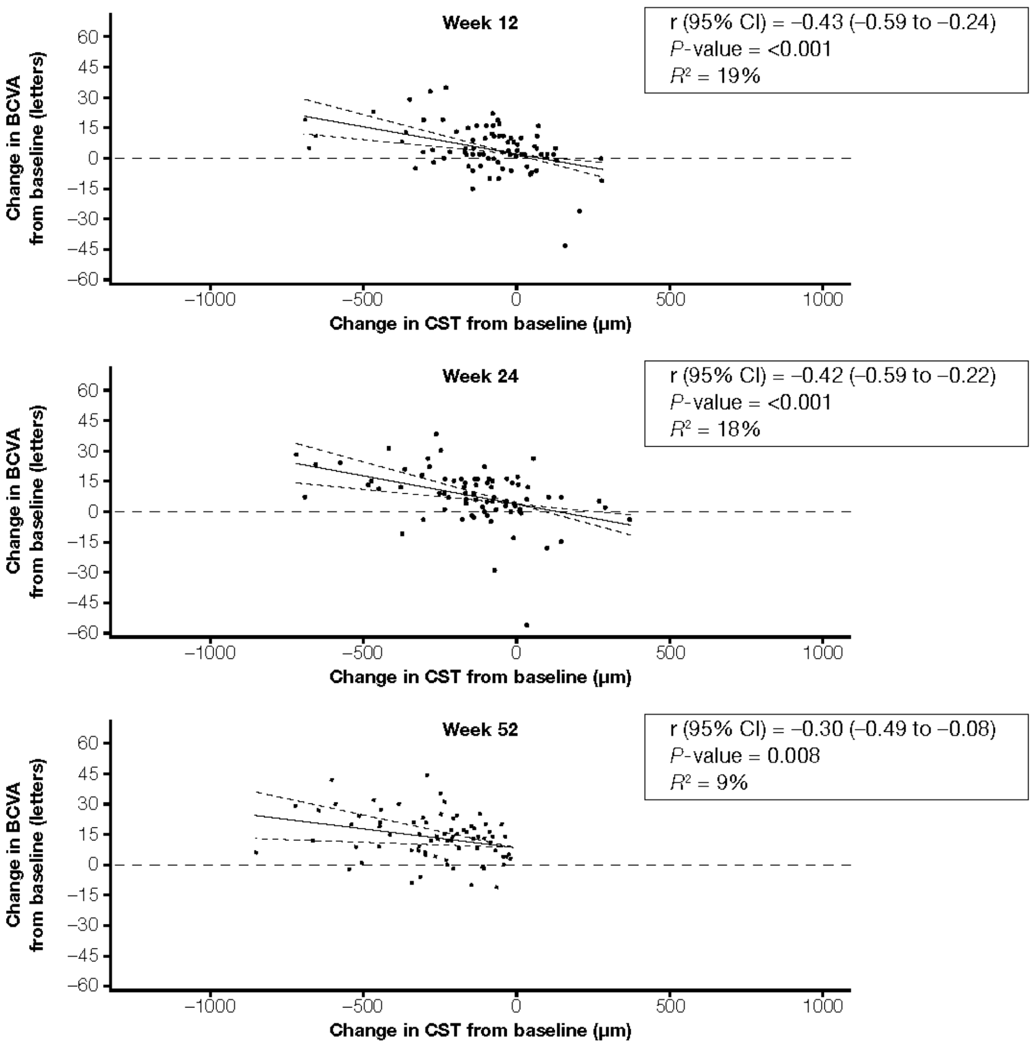

Solid lines indicate the correlation line, and dashed lines indicate the 95% CIs. In VIBRANT, study participants with macular edema secondary to BRVO received either laser at baseline or IAI 2q4 from baseline through week 24. Both treatment groups received IAI 2q8 from week 24 through week 52. 2q4, 2 mg every 4 weeks; 2q8, 2 mg every 8 weeks; BCVA, best-corrected visual acuity; CI, confidence interval; CST, central subfield thickness; IAI, intravitreal aflibercept injection; r, correlation; R^2, coefficient of determination.
